# Recent Medico-Legal Developments on the Issue of Epilepsy and Driver's License Requirements in the Italian and European Legislation

**DOI:** 10.1155/2019/7127956

**Published:** 2019-09-30

**Authors:** Brenno Mazzariol, Antonella Pastorini, Alessandro di Luca, Natale Mario di Luca

**Affiliations:** ^1^Dipartimento di Scienze Anatomiche, Istologiche, Medico Legali e dell'Apparato Locomotore, Università “La Sapienza”, 00161 Roma, Italy; ^2^Fondazione Policlinico Universitario Agostino Gemelli IRCCS, Università Cattolica del Sacro Cuore, 00168 Roma, Italy

## Abstract

Epilepsy is a condition that comprises a group of neurological disorders characterized by seizures. Forms of epilepsy that produce abrupt bouts that cause lapses in consciousness may pose a major road safety problem for drivers who, while going through a seizure, could seriously harm themselves as well as others. A fundamental strategy for the purpose of reducing the risk of car accidents caused by epileptic drivers is constituted by prevention, in addition to adequate pharmacological therapies. In that respect, forensic medicine plays a pivotal role, since it deals with the set of requirements that must be met by those who have been diagnosed with epilepsy in order to get a driver's license, and with the obligation to signal such individuals to the national Driver and Vehicle Licensing Agency (in Italian: *Motorizzazione Civile*). In that regard, the Italian legislative framework is partly hazy in some respects, which the authors have set out to analyze herein, taking into account recently issued European norms. The aim of this paper was to better understand the current Italian legislation in the matter of epilepsy and driver's license requirements, especially regarding the medical criteria that must be met in order to obtain the driving license. The importance of those criteria is underlined by the fact that they directly influence (and are influenced by) the safety for the drivers and for the persons involved in car accidents. Thus, we can consider the issue not only strictly of medico-legal relevance but also from the standpoint of primary prevention. The analysis was conducted by reviewing the most recent documents of medico-legal relevance, in the light of European Union legislation. The authors have ultimately stressed the need for clearer and straightforward regulations, given that professional liability may arise whenever a driver's license is issued, in disregard of legal norms, to an individual who then causes a road accident.

## 1. Introduction

The epidemiology of epilepsy reflects how it represents one of the most common neurological causes of disability, with an estimated 1% prevalence [1] and an incidence ranging between 40 and 200/100,000 [2] globally, despite the clear difficulty to refer to large-scale sets of data, because of the various and heterogeneous groups of disorders that make up the condition itself, the degree of regional variability, and a dearth of relevant studies on the issue. According to a definition that was issued in 2005 by the International League Against Epilepsy (ILAE), an epileptic seizure is “a transient occurrence of signs and/or symptoms due to an abnormal excessive or synchronous neuronal activity in the brain” [3].

As stated in a 2014 ILAE practical new definition [4], epilepsy is a neurological disease defined by any of the following conditions:

1. At least two unprovoked (or reflex) seizures occurring >24 h apart
2. One unprovoked (or reflex) seizure and a probability of further seizures similar to the general recurrence risk (at least 60%) after two unprovoked seizures, occurring over the next 10 years
3. Diagnosis of an epilepsy syndrome

In accordance with the ILAE Seizure Classification of 2017, it is possible to identify three levels of classification of epilepsy, based on the different clinical environments and resources available [5, 6]. The first one concern is the seizure type: the three main seizure categories have been identified, depending on which brain area is originally involved: generalized onset, affecting circuit functions in both hemispheres of the brain; focal onset, which originates in neuronal networks limited to one cerebral hemisphere or part of it; and also an unknown onset. The second level regards the epilepsy type and includes four categories: generalized epilepsy, focal epilepsies, combined generalized and focal epilepsies, and also an unknown epilepsy group. In any case but the unknown category, the diagnosis is both clinical and instrumental (EEG). Each group is further subdivided according to the manifestation of the disease. It is important to underline that the epilepsy type can be the final diagnosis, when it is not possible to achieve an epilepsy syndrome diagnosis. It adds, in addition to the abovementioned findings, imaging features. This third level of diagnosis has no direct correlation with an etiologic diagnosis, but it serves for the purpose of guiding management.

The disease's pathogenesis is quite complex: the basic mechanism can be conceptualized as a distortion of the normal balance between excitation and inhibition in the brain [7], and the reasons behind such an imbalance are manifold and often hard to properly identify. There are, however, numerous genetic and pathological conditions (e.g., structural alterations or acquired cerebral insults) that can result in a predisposition and various triggering factors (when present at all) such as overexposure to lighting and substance [8, 9] or alcohol abuse.

Countless pharmacological therapies are currently available, and the approach to be taken will depend on the type of seizures: the drug (and therefore the molecular action mechanism) will be chosen according to the clinical manifestations.

According to 2014 ILAE report, epilepsy is considered to be resolved for individuals who either had an age-dependent epilepsy syndrome but are now past the applicable age or who have remained seizure-free for the last 10 years and off antiseizure medicines for at least the last 5 years [6]. It is important to underline that in this case the term “resolved” is not synonymous with “cured”: it means that the person has no longer epilepsy, but there is no guarantee that it will not come back again.

Among the most relevant consequences of epilepsy, which causes lapses in consciousness, there is the risk arising from the operation of a motor vehicle, both for the motorists and third parties who may be affected. In addition to the recent Italian legislative crackdown [10], a pivotal prevention strategy aimed at enhancing road safety has been implemented by lawmakers through the identification of the different types of crisis, as well as the definition of the mechanisms that bring it about [11], the risk assessment for each one of them, and the outlining of licensing requirements. A further relevant aspect that should not be overlooked has to do with the unwanted side effects of seizure medication [12] (or AEDs, antiepileptic drugs). Considering valproic acid, one of the most prescribed drugs worldwide for generalized and focal epilepsy, it must be kept in mind that among the side effects, there are minor neurologic ones, such as tremors, sedation, headache, diplopia, and dizziness [13], which can, even though transient an typical of the early period of administration, affect one's fitness to drive. Another common drug prescribed as therapy against epilepsy, carbamazepin, can provoke in the early stages drowsiness, dizziness, and diplopia [14], but even other molecules commonly used for the treatment of the disease can cause neurological effects (such as somnolence, slowed speech and psychomotor function, and mydriasis) that somehow interfere with one's ability to drive: those AEDs are phenytoin, gabapentin, topiramate, lamotrigine, ethosuximide, and others [15]. It has also been found [16] that in car accidents, the collision rate is increased to 97% in association with anticonvulsants. A recent cohort study conducted in Sweden [17] found that epilepsy was associated with a 37% increased risk (hazard ratio (HR) 1.37; 95% confidence interval (CI) 1.29-1.46) of serious traffic accidents over 7 years of “epilepsy” vs. “controls”; however, they also found that there was no significantly increased risk of serious traffic accidents with AEDs.

Most epilepsy-centered studies are cohort studies, based on medical and government databases or questionnaires compiled by individuals with an epilepsy diagnosis [12], although such studies report that people with epilepsy are only moderately more likely to be involved in a traffic accident than people who do not have the condition; the degree of severity of damage in accidents involving people with epilepsy is somewhat higher. A 2015 report by European Road Safety Observatory [18] found that the relative risk of being involved in road accidents due to epilepsy or other seizures is 1,84 (statistically significant at a level of *α* < 0.05).

Studies that have taken into account self-reports from people with epilepsy seem to point to a lower risk in those experiencing auras, because of the warning signs prior to the seizure itself.

As for patients who have undergone surgery to treat refractory forms of epilepsy, a recent report [19] from Britain posits that for individuals with COSY (chance of a seizure in the next year) index below 20%, the risk of causing accidents is lower than population groups such as those aged <25 or >75 years. The same study suggests that a COSY well below 20% has been recorded in patients who have been entirely seizure-free for 1, 2, and 3 years, as well as in patients who had not experienced seizures with loss of consciousness for at least one year.

A legislative overview shows a broad range of driving regulations that apply to people with epilepsy seeking to get or renew their driver's licenses, whereas in Italy, such norms have been profoundly amended over the years, often in an apparently inconsistent fashion. Given how essential driving regulation criteria are, it is of utmost importance to thoroughly peruse all related norms, dispel any doubt or uncertainty as to their interpretation, and highlight possible ethical or moral quandaries, should they arise.

### 1.1. Assessing Fitness to Drive for People with Epilepsy

In several countries all over the world, getting a driver's license requires meeting a clearly defined set of psychological and physical standards, to be documented in writing: for those suffering from epilepsy, such certification may have to be provided by a doctor or produced and signed by the candidates themselves.

In the United States, each state regulates driver's license eligibility of persons with certain medical conditions [20]. Still, the most common standards that people with epilepsy have to meet are a seizure-free interval (of various durations, usually between 3 and 12 months [21]) and a submission of a medical evaluation, documenting their ability to safely drive. Moreover, the periodic submission of medical reports is required in some states for a specific period of time, whereas in others, it is only for as long as the individual retains the license.

In the European Union, directive 2006/126/CE, later amended, has been issued in an effort to make legislation throughout EU member states uniform in terms of outlining common standards while issuing driver's licenses, which are mutually recognized [22]. Specifically, when it comes to the medical standards to be met, epilepsy is mentioned in Annex III, Subsection 12 of the said directive. European lawmakers have characterized epilepsy as “the manifestation of two or more seizures, less than five years apart from each other” and described a provoked epileptic seizure as having “a recognisable causative factor that is avoidable,” subdividing drivers into two groups, depending on what type of vehicle for which the license is required: Group 1 refers to categories A, A1, A2, AM, B, B1, and BE; Group 2 comprises categories C, CE, C1, C1E, D, DE, D1, and D1E.

In accordance with the category for which the license is required, more or less restrictive criteria have been defined (for Group 2 or Group 1, respectively). At any rate, irrespective of the risk assessment associated with any given clinical condition and regardless of the seizure-free time span (with or without the administration of seizure medication), a fundamental point is found in Annex III, Subsection 12.1, which states “A license may be issued or renewed subject to an examination by a competent medical authority and to regular medical check-ups. The authority shall decide on the state of the epilepsy or other disturbances of consciousness, its clinical form and progress (no seizure in the last two years, for example), the treatment received and the results thereof.” That evaluation has therefore to be made by a neurologist, and the results have to be notified to the authority in charge of issuing the license.

Despite the abovementioned European directive, there are still fundamental differences among member states, particularly in terms of how the authorities are informed of the condition. Several research studies from the United States [23] and Greece [24] have highlighted the risk of information being withheld by patients with epilepsy who provided self-reports.

### 1.2. Italian Regulatory Framework

In Italy, the current legislation that sets driving standards has been outlined in Legislative Decree no. 59, 18^th^ April 2011, followed by regulatory guidelines issued by the Ministry of Health on 25^th^ July 2011. The former document [25] came from the adoption on the part of Italy of EU directives 2006/126 and 2009/113; the latter [26] was designed to lay out a set of operational indications pertaining to driving eligibility requirements, including those that individuals with epilepsy must meet.

It would be safe to assume, by looking at those laws, that in order to be declared legally fit to drive, people with epilepsy need to meet a set of requirements to be verified by Regional Medical Boards.

One of the most controversial traits of the Italian legislation has surfaced from a Ministry of Transportation Decree, issued on 30^th^ November 2010 [27], in turn spawned by the adoption of European directive 2009/112/CE. Such a decree clearly states in Annex III, Subsection 2 that “An obligation exists for any hospital, medical, welfare, or insurance institutions, administrations and bodies to report those suffering from epilepsy to the National Bureau of Civil Transportation (the department of motor vehicles, i.e. the Italian licensing authority) once such a condition has been detected and verified (for reasons having to do with the granting of exemptions, disability payments or medico-legal services), so that proper restrictions may be put in place when issuing or renewing the licenses for such patients.”

What appears to be surprising about the EU directive is that it does not mention the obligation to report, which is instead mentioned in the Italian Decree: it is therefore an innovative aspect, introduced by the Italian lawmakers.

As far as the law itself, there is arguably a certain degree of haziness in the interpretation of the wording itself: the “Bodies” and “Administrations” are not specifically mentioned by name, except for a somewhat generic attribution of services which they provide to the individual (social, administrative, welfare-related, or insurance-related). Undoubtedly, a more wide-ranging interpretation of such a norm would lead to health care providers who operate within the National Health Care Service (including general practitioners) being viewed as “representatives” of a given institution (e.g., the National Health Care Service itself), thus bound to report patients with epilepsy to the civil transportation authority, which is in charge of issuing the licenses. Following that characterization, however, doctors who privately practice medicine would be exempted from the duty to report. Another possible and less strict way of construing the piece of legislation is that health care service providers should not be bound to report, whereas such obligation would be limited to forensic medical services that are public in nature.

The broader interpretation of the law clearly entails the involvement of doctors who perform diagnostic or therapeutic activities within the Italian Public Health Care Service in the reporting of patients with epilepsy. Such a scenario, however, would undoubtedly lead to a deterioration and diminution of the trust-based doctor-patient relationship. It behooves one to bear in mind that such a relationship is enshrined in Article 9, Chapter III of the 2014 Italian Code of Medical Ethics [28] (last amended 15^th^ December 2017), in which it is clearly spelled out, in regard to confidentiality, that “doctors are not at liberty to divulge any information that were given to them by their patients, or that they may have learned in the exercise of their profession”; furthermore, the current wording in article 9 has been developed in response to the enactment of law no. 675, in 1996, which has engendered the Italian Authority for the Protection of Personal Data.

The onus on doctors to report epilepsy cases cannot be deemed to be a mere medical ethics issue. First and foremost, in Italy, doctors may be criminally convicted for manslaughter if they acted in conflict with cautionary rules (i.e., rules that have been put in place in order to prevent accidents), thus causing someone's death, whether the deceased was their patient or not. Hence, doctors may be convicted if their neglectful conduct enabled someone, generally unfit to drive, to obtain a driver's license and that led to an accident where either the patient or a third party was injured or died. In Italy, a 2017 piece of legislation, law no. 24, has made it clear that contractual liability is such only if doctors and patients formally enter into a contractual agreement for the provision of health care services. The damaged party can therefore be awarded compensation after proving the damage and the termination of their contract with the doctor. On the other hand, in order to avoid having to pay compensatory damages, doctors need to prove that they operated appropriately and that the damage would have occurred even if all cautionary rules had been complied with. If no contract had ever been signed between the doctor and patient, the physician has noncontractual liability: i.e., in order for the compensation to be granted, claimants will need to prove the damage, a breach of law, or cautionary rules on the part of their doctors and a causal relationship between such a violation and the damage itself [29].

Secondly, it is reasonable to assume that such a mandate would lead patients to be less open and maybe even to omit relevant information about their health conditions in order to avoid being reported. Such a scenario would certainly constitute a potentially serious hazard for patients, since an untruthful or neglectful attitude towards their doctors may cause the definition of a therapeutic pathway to be ineffective. It is also necessary to consider that there are no grounds to prove that mandatory reporting can actually be effective in reducing the incidence of road accidents [30] involving people with epilepsy.

A further potentially harmful aspect, which may stem from the broader interpretation of the law, pertains to the fact that only doctors who do not practice in public health care facilities, or in private clinics not affiliated with it, would be exempted from the duty to report, which would give rise to unequal treatment: those who can afford private clinics and doctors could avoid coming into contact with facilities and physicians bound to report (such as welfare agencies, to which low-income people turn for benefits), whereas low-income people would be in no condition to do that [31].

## 2. Conclusions

In light of the various studies that have been conducted nationally and internationally [32] on the impact of mandatory reporting by physicians on the risks of accidents in drivers with epilepsy, no data ultimately proved that such mandatory reporting rules could reduce the risk of car accidents. Furthermore, concerns about the impact of epilepsy on driving seem to be overrated compared to other medical and nonmedical risks, even though seizures undoubtedly pose a higher risk of causing accidents, compared to the normal population.

Considering that the most controversial point about the adoption of the EU directive is the duty to report individuals with epilepsy who seek a driver's license, it would be advisable to further clarify the terms of involvement for physicians in that respect and reconsider the binding nature of the law itself, in light of the Code of Medical Ethics and the likely repercussions that it might produce, affecting the doctor-patient relationship itself. Lastly, it would be advisable to put an end to, or at least limit, the ambiguity and inconsistencies in the assignment of tasks and roles in Italy, and to map out operative indications with a greater degree of thoroughness and clarity, particularly about the functions of the government bodies and institutions involved. It seems quite clear, in fact, that in case of a car accident caused by an epileptic seizure, the doctors who failed to report the patient's conditions are likely to be held liable for any damage caused to the patient and others by the accident itself. According to Italian law no. 24/2017, both cases would amount to “noncontractual liability,” since patients did not sign any contract with the institution or body where the omitting doctor operated [33]. Even private practitioners, however, should always inform their patients with epilepsy as to the risks that operating a motor vehicle entails in case of an epileptic attack, even in the absence of any duty to report. In those circumstances, “contractual liability” arises, which means that the onus is on physicians to prove that they thoroughly informed their patients [34].
